# Expanding the Clinical Spectrum of *LONP1*-Related Mitochondrial Cytopathy

**DOI:** 10.3389/fneur.2019.00981

**Published:** 2019-10-04

**Authors:** Fady Hannah-Shmouni, Lauren MacNeil, Lauren Brady, Mats I. Nilsson, Mark Tarnopolsky

**Affiliations:** ^1^Clinical Biochemical Genetics, The Hospital for Sick Children, University of Toronto, Toronto, ON, Canada; ^2^Section on Endocrinology and Genetics, National Institute of Child Health and Human Development, National Institutes of Health, Bethesda, MD, United States; ^3^Division of Pediatric Laboratory Medicine, The Hospital for Sick Children, University of Toronto, Toronto, ON, Canada; ^4^Department of Laboratory Medicine and Pathobiology, University of Toronto, Toronto, ON, Canada; ^5^Department of Pediatrics, McMaster University, Hamilton, ON, Canada

**Keywords:** LONP1, mitochondrial cytopathy, granular bodies, whole exome sequencing, electron microscopy

## Abstract

Pathogenic variants in the *LONP1* gene have been associated with CODAS syndrome (Cerebral, Ocular, Dental, *A*uricular, and *S*keletal Anomalies Syndrome). A recent report identified the first newborn case with *LONP1*-related mitochondrial cytopathy due to a compound heterozygous pathogenic variant in *LONP1* without features of CODAS. The proband had manifested with severe congenital lactic acidosis and profound multiple respiratory chain complex activity deficiencies associated with the quantitative loss of mtDNA copy number in muscle. A subsequent report identified two siblings with regression during infancy, profound hypotonia and muscle weakness, severe intellectual disability, progressive cerebellar atrophy, where muscle biopsy showed an electron dense mitochondrial inclusions without ragged-red fibers and normal electron transport chain enzyme activities. Here, we report an additional case of autosomal recessive mitochondrial cytopathy due to a homozygous missense variant in *LONP1* that was identified on whole exome sequencing (c.810G > A; p.D463N). The proband, a 20-year-old male born to consanguineous parents, presented with global developmental delay, emotional outbursts, speech and swallowing difficulties, hypotonia, and ataxia since childhood. Muscle biopsy showed massive granular bodies, increased oxidative stress, and autophagic block and reduced mitochondrial state 3 respiration. We have identified another case of *LONP1*-related mitochondrial cytopathy further confirming a neurological phenotype without CODAS features.

## Introduction

*LONP1* is a lon protease with substrate selectivity, which removes a variety of oxidatively damaged proteins in the mitochondria (1–3). Moreover, *LONP1* is necessary for mitochondrial proteostasis and gene expression maintenance (4). Thus, defects in *LONP1* leads to accumulation of oxidatively damaged proteins, causing mitochondrial specific proteotoxicity (5). In mice, the homozygous deletion of *LonP1* causes early embryonic death (3). In human skeletal muscle, a reduction of *LONP1* activity was shown to have a significantly reduced respiratory chain complex activity, consistent with depletion of mtDNA, which encodes for key components of these respiratory chain complexes (5).

Recently, biallelic pathogenic variants in *LONP1* (19p13.3; OMIM 605490) have been linked to a complex autosomal recessive developmental disorder termed CODAS (Cerebral, Ocular, Dental, Auricular, and Skeletal anomalies; OMIM 600373) syndrome (6, 7). There have also been several case reports of a classic mitochondrial disease phenotype without the classic CODAS features (5, 8). In one report, a compound heterozygous missense variant in *LONP1* (c.1693T> C, p.T565H and c.2197G> A, p.G733L) was identified in a proband who presented in the newborn period with severe lactic acidosis, muscle weakness, and brain MRI typical of Leigh syndrome (5). Muscle biopsy revealed profound multiple respiratory chain complex activity deficiencies associated with a reduction of mtDNA copy number in muscle (5).

In 2017, a group from Japan identified a compound heterozygous variant in *LONP1* on whole exome sequencing in a 12-year-old male with atypical CODAS (9). His manifestations included severe intellectual disability, congenital bilateral cataracts, spasticity, hypotonia, motor regression, and progressive cerebellar atrophy with hyperintensity of the cerebellar cortex on MRI (9). Muscle biopsy was not performed; therefore, it is unclear if this case represented a *LONP1*-related mitochondrial cytopathy. In 2019, a detailed report identified two siblings from a consanguineous family with regression during infancy, profound hypotonia, and muscle weakness, severe intellectual disability and progressive cerebellar atrophy on brain imaging due to a novel homozygous missense *LONP1* variant (c.2282 C > T, p.P761L) (8). Their muscle biopsy revealed scattered cytochrome *c* oxidase-negative staining with electron dense mitochondrial inclusions, no ragged-red fibers (which are commonly observed in mitochondrial cytopathies), and normal activities of all respiratory chain complexes. Additionally, the investigators demonstrated decreased pyruvate dehydrogenase (PDH) activity and elevated intracellular lactate levels, which was caused by increased phosphorylation of E1α (8).

Here, we present a patient with a classical mitochondrial cytopathy due to a homozygous missense variant in *LONP1* identified on whole exome sequencing and expand on the clinical spectrum of *LONP1*-related mitochondrial cytopathy. We demonstrate the presence of massive globular intra-mitochondrial inclusions within skeletal muscle, which represents multifocal electron densities likely reflective of protein that are not broken down through proteases and consistent with *LONP1* deficiency. Additionally, we show an impairment of state 3 respiration capacity which has not been previously reported in this condition.

## Materials and Methods

### Case Report

The proband, a 20-year-old male, was referred to the Neurometabolic Clinic at McMaster University for investigation in the context of gross developmental delay, emotional outbursts, speech and swallowing difficulties, hypotonia, and ataxia since childhood. He was born at term following an uncomplicated pregnancy (birth weight 3,200 g) and developed appropriately until 8 months of age, where he had developmental regression following a mild traumatic brain injury. Axial hypotonia was noted at 1 year of age. He had delays in his walking, started to cruise at 3 years of age and was non-verbal. During his early adulthood, his symptoms progressed, with severe generalized slowing of movements, muscular fatigue, and swallowing difficulties. Currently, he spends most of his time in his wheelchair but walks on occasion with one person assistance. There were no ocular, dental, auricular, or skeletal anomalies identified by clinical examination and targeted X-ray evaluation. His parents are double first cousins from Pakistan with an unremarkable medical or family history. The other family members do not report a history of developmental delay or neurological features.

The proband's physical examination was abnormal. He appeared short and cachectic; he was 50.5 kg, 161.5 cm tall and used a wheelchair for ambulation. He was unable to sit independently. There was evidence of cognitive impairment and non-verbal vocalization, including screams. He would occasionally swat at the examiner. Cranial nerve examination demonstrated horizontal and rotatory nystagmus without ptosis, cataracts, or retinopathy. He had a right eye esotropia. Cerebellar examination demonstrated ataxia and bilateral coarse hand tremors. Lower cranial nerves and hearing were normal. Motor examination demonstrated generally reduced muscle bulk, paratonia, with grade 3–4-/5 muscle weakness. He had normal muscle stretch reflexes in the upper extremities and absent in the lower extremities with downgoing toes. His serial brain MRI's demonstrated progressive cerebellar and vermian atrophy with increased FLAIR signal in cerebellum and periventricular white matter. His echocardiogram, nerve conduction study, *POLG* sequencing, serum lactate, and karyotype were normal.

### Whole Exome Sequencing

After receipt of written informed consent, additional biologic specimens were obtained from the proband to further investigate the possibility of a mitochondrial disorder. Additionally, the proband was enrolled in the Care4Rare (C4R) initiative, a pan-Canadian collaborative team of clinicians, bioinformaticians, scientists, and researchers, focused on improving the care of Rare Disease patients in Canada and around the world (www.care4rare.ca), where research whole exome sequencing was performed from DNA extracted from blood given the suspicion for an autosomal recessive condition. Whole exome sequencing was performed according to their standard approach as previously described (10). DNA sequencing of family members was not available.

### Muscle Biopsy

A repeat muscle biopsy was performed in our institution from the *Vastus lateralis* using a 5 mm modified Bergstrom needle and assessed at the light and electronic microscopic level, as previously described (11). Electron microscopy samples were plastic embedded and ultrathin sections were sent for electron microscopic evaluation, as previously described (12). The maximal activity of several respiratory chain enzymes (complex I + III, II + III, COX, and citrate synthase) were completed from frozen muscle, as previously described (13). High resolution respirometry measurements on freshly isolated and muscle fiber bundles was completed using an Oroboros instrument (Oxygraph-2K, Innsbruck, Austria) and previously described methodology (14). Light microscopic evaluation on cryosections at 7 μm were completed for cytochrome *c* oxidase, succinate dehydrogenase, modified Gomori trichrome, hematoxylin and eosin, oil red-O, and NADH.

### Histopathological and Biochemical Analyses of OXPHOS Function in Muscle

As previously described by Krieger et al. (15) and Cogswell et al. (16), 20 mg of *Vastus lateralis* (VL) muscle was minced, trimmed of fat and connective tissue, weighed, and homogenized in 500 μL of ice-cold buffer (100 mM KCl, 10 mM MOPS, 5 mM EDTA, 5 mM HNa2O4P, Roche complete EDTA-free protease inhibitor cocktail, pH 7.4) with a Polytron homogenizer (Pro Scientific) for 10 s at the halfway setting. Half of the muscle homogenate (250 μL) was suspended in 100 μL RIPA buffer (Thermo Scientific) with protease inhibitors (Roche), vortexed, and frozen at −80°C for mixed protein analyses. The remainder was centrifuged for 10 min at 800 g and the resulting supernatant was centrifuged for 14,000 g for 20 min to obtain subsarcolemmal (SS) mitochondria. The SS mitochondrial pellet was then re-suspended in 50 μL of ice-cold buffer (100 mM KCl, 10 mM MOPS, Roche protease inhibitors, pH 7.4) and frozen at −80°C for SS mitochondrial analyses.

Mixed protein and SS mitochondrial samples were then thawed and suspended in 6X Laemmli loading buffer, heated for 10 min at 37.5°C, and loaded in equal amounts onto 4–20% Criterion TGX pre-cast gels (BIO-RAD). Following electrophoresis at 110 V for 2 h (BIO-RAD), the proteins were transferred to 0.2 μM nitrocellulose membranes using the Trans-Blot Turbo Transfer System (2.5 A and 25 V for 7 min; BIO-RAD). Equal loading was verified by the Ponceau S stain and membranes were blocked in 5% BSA (4-HNE) or 2.5% milk (MS 604) for 1 h at RT, followed by overnight incubation with primary antibodies against 4-hydroxynoneal (1:1,000 4-HNE in 5% BSA; Abcam) or total OXPHOS (1:1,000 MS 604 in 2.5% milk; Abcam) at 4°C. Secondary antibody incubation was performed using anti-mouse or anti-rabbit antibodies (715-035-150 and 711-035-152, respectively; Jackson Laboratories) at 1:20,000 dilution in 5% BSA (4-HNE) or 2.5% milk (MS 604) for 1 h at RT. Membranes were then developed with enhanced chemiluminescence (Clarity Western ECL; BIO-RAD), images obtained using a ChemiDoc MP Imaging System (BIO-RAD), and optical density quantified by standard methods (ImageJ software, National Institutes of Health, USA). The 4-HNE is a commonly used marker of oxidative damage and correlates well with other markers of OXPHOS damage across various tissues, such as nitrotyrosine and protein carbonyls.

### Fibroblast Studies

Lactate/Pyruvate (L/P) ratio was determined using the protocol outlined previously (17). Briefly, confluent skin fibroblast cultures were drained of culture medium, 1 mL of sterile phosphate buffered saline (PBS) added to the plate and cultures incubated at 37°C for 1 h to deplete glycogen reserves. PBS was aspirated, 1 mL of 1 mM glucose in PBS added and incubated at 37°C for 1 h. Immediately after incubation, 0.05 mL of 1.6 M perchloric acid was added and supernatant isolated. After centrifugation supernatant was assayed for lactate and pyruvate activity and L/P ratio calculated.

Mitochondria were isolated from cultured skin fibroblasts and activity rotenone-sensitive NADH-cytochrome *c* reductase spectrophotometrically by the method of Moreadith et al. (18). Whole cell fibroblast cytochrome *c* oxidase and citrate synthase activity were determined spectrophotometrically as described by Glerum et al. (19) and Shepherd and Garland (20). Enzyme activities were normalized to protein content determined by the Lowry method and expressed as nmole/min/mg.

Fibroblast PDH total enzyme activity was measured in both the native and DCA-activated state (21). Confluent skin fibroblast cultures were harvested and suspended in two sterile PBS solutions, with or without dichloroacetic acid (DCA). Lysed cells were incubated in buffer containing [1-^14^C] pyruvate (Amersham Biosciences, Arlington Heights, IL) at 37°C for 10 min. The reaction was stopped and radioactive CO_2_ released by PDH activity counted on a scintillation counter. Enzyme activities were normalized to protein content determined by the Lowry method and expressed as nmole/min/mg.

## Results

### A Diagnostic Muscle Biopsy Reveals Multiple OXPHOS Deficiencies and Large Globular Inclusions

Muscle histopathology, histochemistry, and mitochondrial electron transport chain activities were initially investigated prior to our consultation. This demonstrated increased sub-sarcolemmal succinate dehydrogenase staining and occasional cytochrome *c* oxidase (COX) negative fibers, without ragged red fibers, and globular inclusions on electron microscopy. mtDNA sequencing and CoQ10 levels from muscle tissue were both normal.

**Figure 1:**
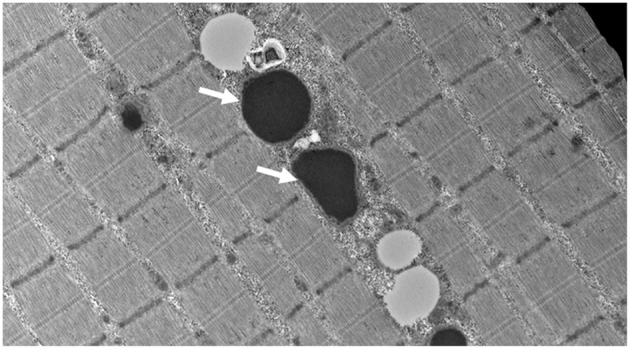
Muscle biopsy ultrastructure demonstrating intra-mitochondrial globular inclusions (arrows) (15,000 X).

At our center, several experiments were performed. The frozen skeletal muscle biopsy on the hematoxylin and eosin sections showed skeletal muscles with fibers largely in transverse orientation with a population of smaller and atrophic fibers. There was mild increase in the interstitial endomysial fibrous tissue and internalized nuclei. The modified Gomori trichome did not show any abnormal inclusions or rimmed vacuoles, and there were no ragged red fibers. COX was normally reactive in most fibers; however, there were scattered populations of very weak/negative fibers. NADH was unremarkable with no cores or abnormal inclusions. The ultrastructural examination showed globular inclusions representing multifocal electron densities, likely reflective of protein this is not broken down through proteases (Figure 1).

**Figure 2:**
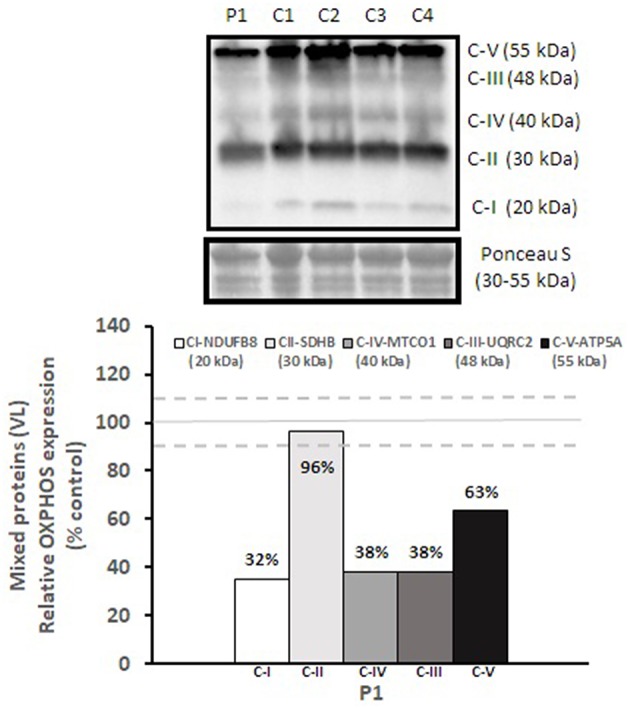
OXPHOS expression in *vastus lateralis* muscle. Complex I, II, III, IV, and V expression in *vastus lateralis* muscle were normalized to total protein (Ponceau S) and presented as a percentage of healthy, gender, and age-matched controls (*N* = 4). Solid gray line indicates normal protein levels (e.g., control mean is 100%) and dashed lines are bidirectional standard errors calculated from the absolute values, control mean, and *N*.

**Figure 3:**
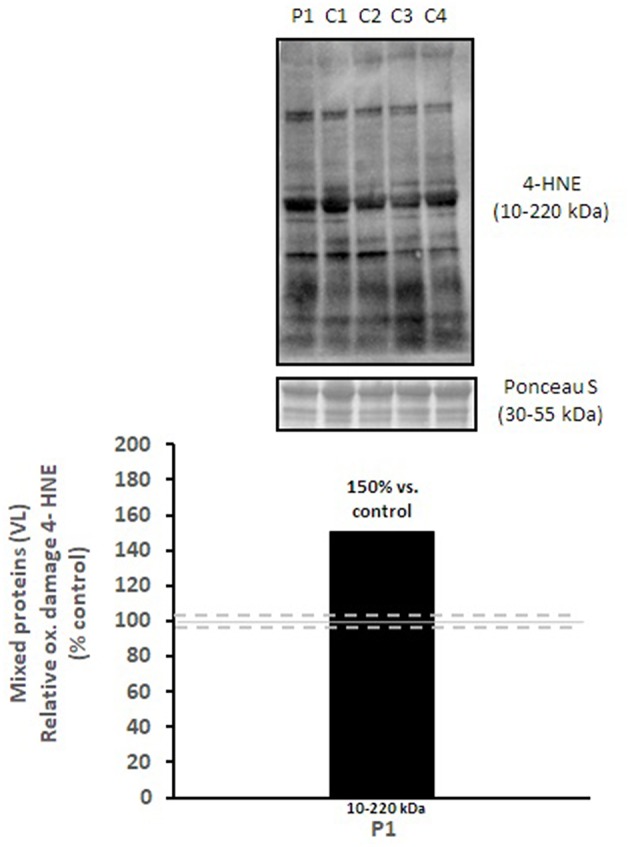
Oxidative damage of mixed proteins in *vastus lateralis* muscle. 4-HNE levels of mixed proteins in *vastus lateralis* muscle were normalized to total protein (Ponceau S) and presented as a percentage of healthy, gender, and age-matched controls (*N* = 4). Solid gray line indicates normal 4-HNE levels (e.g., control mean is 100%) and dashed lines are bidirectional standard errors calculated from the absolute values, control mean, and *N*.

**Figure 4:**
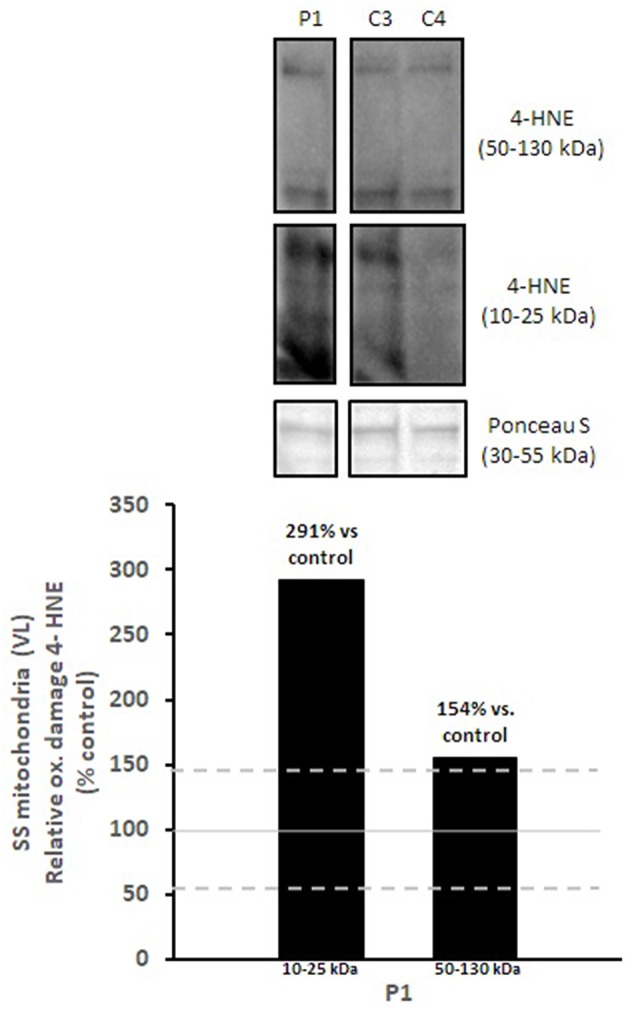
Oxidative damage of subsarcolemmal mitochondria in *vastus lateralis* muscle. 4-HNE levels of subsarcolemmal mitochondria isolated from *vastus lateralis* were normalized to total protein (Ponceau S) and presented as a percentage of healthy, gender, and age-matched controls (*N* = 2). Solid gray line indicates normal 4-HNE levels (e.g., control mean is 100%) and dashed lines are bidirectional standard errors calculated from the absolute values, control mean, and *N*. The samples are not biological replicates *per se* since each lane is a unique subject, and the standard error of the mean for the healthy controls is what is reflected in the lines to use as a comparison to the patient.

Mitochondrial respiratory chain enzyme activities were lower than controls; however, when expressed relative to citrate synthase they were normal or above normal (Table 1); however, high resolution respirometry showed significant defects (e.g., >2 SD below control means) for state 2 and complex I supported state 3 respiration (Table 2). OXPHOS expression showed an ~50% reduction in enzyme complexes that have some mtDNA encoded subunits (Figure 2). Although the maximal activity of the respiratory chain enzymes were normal (Table 1), the protein content as determined by Western blot were uniformly low with the exception of complex II (Figure 3). Oxidative damage of mixed proteins and subsarcolemmal mitochondria in *Vastus lateralis* muscle were evident (Figure 4). Taken together, this biopsy was consistent with partial heteroplasmic-type COX deficiency consistent with a mitochondrial cytopathy with associated mild myopathic changes.

**Table 1:** Mitochondrial enzyme activity from muscle.

**Enzyme test**	**Result** **U/mg protein**	**Mean**	**SD**	**%**	**% relative to CS**
Cytochrome *c* oxidase (COX)	26	39	24	66	150
Complex I	12	24	18	50	114
Complexes I + III	25	32	17	79	180
Complex II	12	18	9	69	157
Complexes II + III	7	20	7	36	82
Complex III	113	87	61	130	296
Citrate Synthase	164	374	169	44	–

*The measurements were performed in one biological sample, and the mean and standard deviation (SD) refer to technical replicates*.

**Table 2:** Mitochondrial high resolution respirometry from muscle.

**Absolute**	**Result** Pmol/(s^*^mg ww)	**Mean**	**SD**	**%**	**% relative to CS**
State 2	0.2	9	2	2	5
State 3 (CI)	10	36	14	28	64
State 3 (CI+II)	29	83	23	35	80
State 3 (CII)	23	59	15	38	87
CIV (TMPD)	217	305	56	71	162

### Results of Fibroblast Studies

The analysis of confluent skin fibroblast studies (Table 1) showed a normal L/P ratio and mitochondrial enzyme activity. Pyruvate dehydrogenase activity was mildly lower than control ranges in the native form, but normal for DCA activated activity (Table 3).

**Table 3:** Analysis of confluent skin fibroblast cultures.

	**Ratio**	**Reference interval**
Lactate/pyruvate ratio (L/P)	11.9	9.6–26.5
	**Enzyme activity (nmole/min/mg)**	**Reference Interval (nmole/min/mg)**
**Mitochondrial enzyme activity**		
NADH: Cytochrome *c* reductase, rotenone sensitive (complex I + III)	41.0	23.6-77.8
Cytochrome oxidase (complex IV)	5.3	3.5–12.5
Citrate synthase (CS)	42.3	36.5–80.3
**Pyruvate dehydrogenase activity**		
Pyruvate dehydrogenase (PDH) (native)	0.34	0.46–1.60
Pyruvate dehydrogenase (PDH) (dichloroacetate activated)	1.05	0.87–2.33

### Whole Exome Sequencing Identifies a Novel Homozygous Missense Variant in *LONP1*

Analysis for autosomal recessive variants in nuclear genes encoding mitochondrial-targeted proteins was prioritized. A novel homozygous missense variant in the *LONP1* gene (chr19:g.5700919C>T, NM_001276480.1: exon9, c.810G>A, p.D463N) was detected, predicted to be located within a highly conserved domain. This variant was not present in ExAC (Exome Aggregation Consortium, exac.broadinstitute.org), gnomAD (The Genome Aggregation Database, gnomad.broadinstitute.org) or ClinVar (ncbi.nlm.nih.gov/clinvar). Thus, given that the variant was homozygous and not present in the databases, we did not need to phase the variant in parents. Collectively, the results of his genetic testing with supportive evidence of intra-mitochondrial accumulation of oxidatively damaged proteins and respiratory chain deficits in complexes that included mtDNA sub-units helped establish the diagnosis of *LONP1*-related mitochondrial cytopathy.

### Follow Up

He was started on a mitochondrial cocktail, which consisted of alpha lipoic acid, co-enzyme Q10, creatine monohydrate, and vitamin E (22), with no clinically significant improvement in symptoms reported; however, his neurological symptoms have not progressed to date.

## Discussion

In this investigation, we describe an additional case of a severe mitochondrial cytopathy resulting from a homozygous missense variant in *LONP1*, and show an expected finding of massive intra-mitochondrial globular inclusions on muscle biopsy, likely representing protein that is not broken down through proteases, in keeping with *LONP1* deficiency (Figure 1). This mitochondrial defect did not result in the canonical histological (COX-negative, RRF, paracrystalline inclusions) or enzymatic (frozen tissue maximal activity of respiratory chain enzymes) features of a mitochondrial cytopathy; yet, the finding of large intra-mitochondrial electron densities and very significant reductions in high resolution respirometry determined activities were defining, and in keeping with the muscle findings of a previous report (8). Additionally, we showed a decrease in all mitochondrial electron transport chain subunits, and a decrease in native PDH activity. The PDH dysfunction seen in *LONP1*-related mitochondrial cytopathy is due to increased levels of the phosphorylated E1α subunit of PDH, which inhibits enzyme activity (8). These finding adds to the current knowledge from the few reports of *LONP1*-related mitochondrial cytopathy and suggests that a varying muscle tissue mitochondrial pathology and electron transport chain subunits activity exists in this condition (5).

When compared to past decades, muscle biopsy has fallen off as gold standard for the evaluation of mitochondrial cytopathies (23). Current consensus recommendations for the investigation of mitochondrial cytopathies place mtDNA or nuclear-based sequencing as first line tests (24, 25). In this report, we describe investigations for mitochondrial cytopathy that emphasize the value of a muscle biopsy, especially with electron microscopy and high resolution respirometry. Thus, analysis of muscle tissue may provide important diagnostic information for some mitochondrial cytopathies, including *LONP1*-related disorders. An issue with next generation sequencing and whole exome sequencing is that several potential candidate pathogenic variants are often called and the muscle biopsy information in this case was biologically consistent with the expected pathological effects of a pathogenic variant in *LONP1* and allowed for a tight genotype-phenotype relationship to be established.

*LONP1* is a lon protease which removes oxidatively damaged proteins in the mitochondrion, defects of which leads to accumulation of damaged proteins, causing mitochondrial specific proteotoxicity (1–3). To date, there are a few case reports of a *LONP1* manifesting with a mitochondrial disease phenotype (5, 8). Other reports of recessively inherited variants in *LONP1* have been described in relation to CODAS syndrome (6, 7, 9), which could be distinguished from classical mitochondrial diseases by its distinctive clinical anomalies. However, patients with CODAS manifest with a varying clinical spectrum that may mimic that of a classical mitochondrial disease, including but not limited to, short stature, ptosis, hypotonia, and intellectual disability (6, 7, 26). Our findings are unique in that the muscle biopsy findings were consistent with a mitochondrial cytopathy resulting from an intra-mitochondrial proteolytic defect, showing lower state 3 respiration and some mitochondrial proteins as compared to controls. Nimmo et al. showed reduced fibroblast PDH activity due to increased phosphorylation of E1α because of LonP1-p.P761L, that normalized with an inhibitor (8). Our findings of a lower native PDH activity was in keeping with their report (8); however, we did not find a reduction of DCA activated PDH activity, perhaps reflecting a less severe impact of the *LONP1* deficiency upon PDH activity induced by the specific variation change described herein (p.D463N).

It is important to note that our patient showed none of the dysmorphic clinical features of CODAS despite targeted phenotyping including X-rays for the classical skeletal abnormalities. Currently, it is unclear why some pathogenic variants in *LONP1* lead to CODAS syndrome and others to a neurological predominant mitochondrial cytopathy. It is likely that many of the children with CODAS do have pathological features of a mitochondrial disease but without electron microscopic and high resolution respirometry evaluation of muscle, the defect would likely not be discovered. To better understand the pathological impact of pathogenic variants in *LONP1* in cases of CODAS syndrome (9), our data suggests that a muscle biopsy with electron microscopy and high resolution respirometry should be considered.

In summary, we report an additional case of *LONP1*-related mitochondrial cytopathy with abnormalities on muscle biopsy in keeping with the condition. Although we did not perform functional studies of *LONP1* specific activity for this variant to ascertain its pathogenicity (e.g.,: misfolded protein determination in mitochondrial preprations); the similarity of the clinical phenotype to two other publications, the presence of massive intra-mitochondrial accumulations, and the presence of a defect in state 3 respiration and native PDH activity are strongly supportive of pathogenicity of the p.D463N variant. Furthemore, the finding of muscle biopsy pathology similar to those seen in the two other resports and the dramatic intra-mitochodnrial inclusions support that muscle biopsy analysis with electron microscopy should be completed in cases of CODAS and mitochondrial cytopathy *LONP1* variants.

## Ethics Statement

This study was carried out in accordance with the ethical standards of McMaster University and with written informed consent from the subject's father (power of attorney) who signed the consent. The subject's father gave written informed consent in accordance with the Declaration of Helsinki.

## Informed Consent

Informed consent was obtained from the participant for the publication of this case report.

## Author Contributions

All authors listed have made a substantial, direct and intellectual contribution to the work, and approved it for publication.

### Conflict of Interest Statement

Mr. Dan Wright and family provided funds for the completion of the study and all data was collected in the Corkins/Lammert Neuromuscular and Neurometabolic Center. MT is partially supported by an endowed chair in Neuromuscular Disease and is CEO of Exerkine Corporation, a company working on the discovery of therapies for aging and neuromuscular and neurometabolic disorders including mitochondrial disease. The remaining authors declare that the research was conducted in the absence of any commercial or financial relationships that could be construed as a potential conflict of interest.

## Abbreviations

CODAS syndrome: Cerebral, Ocular, Dental, Auricular, and Skeletal Anomalies Syndrome.

## References

[1] Arnold I Langer T. Membrane protein degradation by AAA proteases in mitochondria. Biochim Biophys Acta. (2002) 1592:89–96. 10.1016/S0167-4889(02)00267-7 12191771

[2] Gerdes F Tatsuta T Langer T. Mitochondrial AAA proteases–towards a molecular understanding of membrane-bound proteolytic machines. Biochim Biophys Acta. (2012) 1823:49–55. 10.1016/j.bbamcr.2011.09.015 22001671

[3] Quiros PM Espanol Y Acin-Perez R Rodriguez F Barcena C Watanabe K. ATP-dependent Lon protease controls tumor bioenergetics by reprogramming mitochondrial activity. Cell Rep. (2014) 8:542–56. 10.1016/j.celrep.2014.06.018 25017063

[4] Zurita Rendon O Shoubridge EA. *LONP1* is required for maturation of a subset of mitochondrial proteins and its loss elicits an integrated stress response. Mol Cell Biol. (2018) 38:e00412–7. 10.1128/MCB.00412-17 30061372 PMC6168981

[5] Peter B Waddington CL Olahova M Sommerville EW Hopton S Pyle A. Defective mitochondrial protease *LONP1* can cause classical mitochondrial disease. Hum Mol Genet. (2018) 27:1743–53. 10.1093/hmg/ddy080 29518248 PMC5932559

[6] Dikoglu E Alfaiz A Gorna M Bertola D Chae JH Cho TJ. Mutations in LONP1, a mitochondrial matrix protease, cause CODAS syndrome. Am J Med Genet A. (2015) 167:1501–9. 10.1002/ajmg.a.37029 25808063

[7] Strauss KA Jinks RN Puffenberger EG Venkatesh S Singh K Cheng I. CODAS syndrome is associated with mutations of LONP1, encoding mitochondrial AAA+ Lon protease. Am J Hum Genet. (2015) 96:121–35. 10.1016/j.ajhg.2014.12.003 25574826 PMC4289676

[8] Nimmo GAM Venkatesh S Pandey AK Marshall CR Hazrati LN Blaser S. Bi-allelic mutations of *LONP1* encoding the mitochondrial *LONP1* protease cause pyruvate dehydrogenase deficiency and profound neurodegeneration with progressive cerebellar atrophy. Hum Mol Genet. (2019) 28:290–306. 10.1093/hmg/ddy351 30304514 PMC6322071

[9] Inui T Anzai M Takezawa Y Endo W Kakisaka Y Kikuchi A. A novel mutation in the proteolytic domain of *LONP1* causes atypical CODAS syndrome. J Hum Genet. (2017) 62:653–5. 10.1038/jhg.2017.11 28148925

[10] Beaulieu CL Majewski J Schwartzentruber J Samuels ME Fernandez BA Bernier FP. FORGE Canada Consortium: outcomes of a 2-year national rare-disease gene-discovery project. Am J Hum Genet. (2014) 94:809–17. 10.1016/j.ajhg.2014.05.003 24906018 PMC4121481

[11] Tarnopolsky MA Pearce E Smith K Lach B. Suction-modified Bergstrom muscle biopsy technique: experience with 13,500 procedures. Muscle Nerve. (2011) 43:717–25. 10.1002/mus.21945 21462204

[12] Devries MC Samjoo IA Hamadeh MJ McCready C Raha S Watt MJ. Endurance training modulates intramyocellular lipid compartmentalization and morphology in skeletal muscle of lean and obese women. J Clin Endocrinol Metab. (2013) 98:4852–62. 10.1210/jc.2013-2044 24081737

[13] Gianni P Jan KJ Douglas MJ Stuart PM Tarnopolsky MA. Oxidative stress and the mitochondrial theory of aging in human skeletal muscle. Exp Gerontol. (2004) 39:1391–400. 10.1016/j.exger.2004.06.002 15489062

[14] Monaco CMF Hughes MC Ramos SV Varah NE Lamberz C Rahman FA. Altered mitochondrial bioenergetics and ultrastructure in the skeletal muscle of young adults with type 1 diabetes. Diabetologia. (2018) 61:1411–23. 10.1007/s00125-018-4602-6 29666899

[15] Krieger DA Tate CA McMillin-Wood J Booth FW. Populations of rat skeletal muscle mitochondria after exercise and immobilization. J Appl Physiol Respir Environ Exerc Physiol. (1980) 48:23–8. 10.1152/jappl.1980.48.1.23 6444398

[16] Cogswell AM Stevens RJ Hood DA. Properties of skeletal muscle mitochondria isolated from subsarcolemmal and intermyofibrillar regions. Am J Physiol. (1993) 264(2 Pt 1):C383–9. 10.1152/ajpcell.1993.264.2.C383 8383431

[17] Robinson BH. Use of fibroblast and lymphoblast cultures for detection of respiratory chain defects. Methods Enzymol. (1996) 264:454–64. 10.1016/S0076-6879(96)64041-5 8965718

[18] Moreadith RW Batshaw ML Ohnishi T Kerr D Knox B Jackson D. Deficiency of the iron-sulfur clusters of mitochondrial reduced nicotinamide-adenine dinucleotide-ubiquinone oxidoreductase (complex I) in an infant with congenital lactic acidosis. J Clin Invest. (1984) 74:685–97. 10.1172/JCI111484 6432847 PMC425222

[19] Glerum DM Yanamura W Capaldi RA Robinson BH. Characterization of cytochrome-c oxidase mutants in human fibroblasts. FEBS Lett. (1988) 236:100–4. 10.1016/0014-5793(88)80293-X 2841159

[20] Shepherd D Garland PB. The kinetic properties of citrate synthase from rat liver mitochondria. Biochem J. (1969) 114:597–610. 10.1042/bj1140597 5820645 PMC1184933

[21] Maj MC MacKay N Levandovskiy V Addis J Baumgartner ER Baumgartner MR. Pyruvate dehydrogenase phosphatase deficiency: identification of the first mutation in two brothers and restoration of activity by protein complementation. J Clin Endocrinol Metab. (2005) 90:4101–7. 10.1210/jc.2005-0123 15855260

[22] Parikh S Goldstein A Karaa A Koenig MK Anselm I Brunel-Guitton C. Patient care standards for primary mitochondrial disease: a consensus statement from the Mitochondrial Medicine Society. Genet Med. (2017) 19:1–18. 10.1038/gim.2017.107 28749475 PMC7804217

[23] Bernier FP Boneh A Dennett X Chow CW Cleary MA Thorburn DR. Diagnostic criteria for respiratory chain disorders in adults and children. Neurology. (2002) 59:1406–11. 10.1212/01.WNL.0000033795.17156.00 12427892

[24] Parikh S Goldstein A Koenig MK Scaglia F Enns GM Saneto R. Diagnosis and management of mitochondrial disease: a consensus statement from the Mitochondrial Medicine Society. Genet Med. (2015) 17:689–701. 10.1038/gim.2014.177 25503498 PMC5000852

[25] Witters P Saada A Honzik T Tesarova M Kleinle S Horvath R. Revisiting mitochondrial diagnostic criteria in the new era of genomics. Genet Med. (2018) 20:444–51. 10.1038/gim.2017.125 29261183

[26] Khan AO AlBakri A. Clinical features of LONP1-related infantile cataract. J AAPOS. (2018) 22:229–31. 10.1016/j.jaapos.2017.10.012 29408517

